# The TCM Prescription Yi-Fei-Jie-Du-Tang Inhibit Invasive Migration and EMT of Lung Cancer Cells by Activating Autophagy

**DOI:** 10.1155/2022/9160616

**Published:** 2022-01-29

**Authors:** Shanshan Wang, Zaichuan Wang, Yinqiu Wu, Chao Hou, Xiaojun Dai, Qingyin Wang, Yongjian Wu, Chao Qian, Xiaochun Zhang

**Affiliations:** ^1^Department of Oncology, Yangzhou Hospital of Traditional Chinese Medicine, (TCM), Yangzhou 225002, China; ^2^The State Administration of Traditional Chinese Medicine Key Laboratory of Toxic Pathogens-Based Therapeutic Approaches to Gastric Cancer, Yangzhou University, Yangzhou 225009, China; ^3^School of Medicine, Yangzhou University, Yangzhou 225009, China

## Abstract

Yi-Fei-Jie-Du-Tang (YFJDT) is a traditional Chinese medicine formulation. Our previous studies have demonstrated that YFJDT can be used to treat non-small-cell lung cancer (NSCLC), but its protective effect against NSCLC and its mechanisms remain unclear. In the present study, we evaluated the protective effects and potential mechanisms of YFJDT on a tumor-bearing mouse lung cancer model and A549 cell model. Tumor-bearing mice and A549 cells were treated with YFJDT, tumors were measured during the experiment, and tumor tissues and cell supernatants were collected at the end of the experiment to assess the levels of autophagy and epithelial-mesenchymal transition (EMT)-related proteins. The results showed that YFJDT treatment reduced tumor volume and mass, increased the expression of the autophagy marker LC3, and inhibited EMT-related proteins compared with the model group. Cell survival was reduced in the YFJDT-treated groups compared with the model group, and YFJDT also reduced the migration and invasion ability of A549 cells in a dose-dependent manner. Western blotting detected that YFJDT also upregulated FAT4 in the tumor tissue and A549 cells and downregulated the expression of vimentin. Meanwhile, apoptosis in both tissues and cells was greatly increased with treatment of YFJDT. We further interfered with FAT4 expression in cells and found that the inhibitory effect of YFJDT on EMT was reversed, indicating that YFJDT affects EMT by regulating FAT4 expression. Taken together, results of this study suggested that the inhibitory effect of YFJDT on EMT in lung cancer tumors is through upregulating FAT4, promoting autophagy, and thus inhibiting EMT in cancer cells.

## 1. Introduction

Lung cancer is currently one of the major malignancies threatening human health, with the highest incidence and mortality rate [1]. Non-small-cell lung cancer (NSCLC) is the most predominant type of lung cancer, and metastasis is its malignant feature and an important factor affecting patient survival and prognosis [2, 3]. How to suppress metastasis is a key breakthrough tool that promote the prognosis of lung cancer patients. Epithelial-mesenchymal transition (EMT) is involved in the invasion and metastasis of lung cancer and plays an important role in them [4, 5]. Recent research studies have shown that autophagy affects the invasion and metastasis of tumor cells by regulating EMT [6, 7]. Thus, modulation of autophagy to inhibit the development of tumor has become a new direction in tumor therapy.

Yi-Fei-Jie-Du-Tang (YFJDT) is a traditional Chinese medicine formula containing Chinese medical plants, namely, *Glehnia littoralis* F. Schmidt ex Miq. (Beishashen), *Ophiopogon japonicus* (L.f.) Ker-Gawl (Maidong), *Pseudostellaria heterophylla* (Miq.) Pax ex Pax et Hoffm. (Taizishen), *Euphorbia helioscopia* L., (Zeqi), *Asarum sagittarioides* C. F. Liang. (Shancigu), *Sarcandra glabra* (Thunb.) Nakai. (Zhongjiefeng), *Ranunculus ternatus* Thunb. (Maozhuacao), Baijiangcan, *Oldenlandia diffusa* (Willd) Roxb. (Baihuasheshecao), and *Agrimonia pilosa* Ledeb. (Xianhecao), which was created by Professor Zhongying Zhou, a master of National Chinese Medical Science and a famous traditional Chinese medicine (TCM) expert, combining the etiology of malignant tumors and his many years of clinical experience [8]. In this decoction, Baishashen, Maidong, and Taizishen have a moistening effect on the lungs; Zeqi, Shancigu, and Maozhuacao could dissolve phlegm and detoxify nodules; Baijiangcan could dispel phlegm and subdues swelling; Baihuasheshecao could clear heat and detoxify the lung; and Xianhecao could nourish deficiency and detoxify the lung. Modern pharmacological studies have shown that all the medicines in this formula have certain antitumor effects [9,10].

Our previous study suggested that YFJDT could inhibit hypoxia-induced invasion, migration, and EMT of A549 cells [8]. The FAT4 gene belongs to the FAT family and is a tumor suppressor [11]. It is expressed at a significantly lower rate than normal tissues in a variety of malignant tumors, including gastric, breast, and liver cancers [12–14]. FAT4 is also a cell adhesion protein that affects the migration of cells. However, it is unclear whether the inhibitory effects of YFJDT on hypoxia-induced invasion, migration, and EMT of A549 cells are related to cellular autophagy as well as FAT4. Therefore, this study aims to investigate the molecular mechanism of the inhibitory effect of YFJDT on the invasion and migration of lung cancer cells *in vitro* and *in vivo* by applying modern molecular biology techniques under the guidance of Chinese medicine theory and taking the autophagy pathway as the entry point. The study will provide a laboratory basis for the clinical application and development of YFJDT.

## 2. Material and Methods

### 2.1. Preparations and Component Analysis of YFJDT

YFJDT is composed of 12 g of Beishashen, 10 g of Maidong, 12 g of Taizishen, 12 g of Shancigu, 15 g of Zeqi, 20 g of Maozhuacao, 20 g of Zhongjiefeng, 15 g of Xianhecao, 10 g of Baijiangcan, and 20 g of Baihuasheshecao. The herbs were purchased from the Chinese Pharmacy of Yangzhou Hospital of Traditional Chinese Medicine affiliated with Nanjing University of Chinese Medicine. The criteria for identifying the quality of the herbs used were in accordance with the 2005 edition of the Chinese Pharmacopoeia (Chinese Pharmacopoeia Commission, Pharmacopoeia of the People's Republic of China, Beijing: People's Medical Publishing House; 2005). Prior to their use in experiments, the herbs were tested for heavy metals, microbial contamination, and residual pesticides, and all results met the safety standards in China. Laboratory personnel were blinded to the identity of the herbs. A trained technician prepared the decoction according to a standardized procedure. The herbs were steeped in double-distilled water for 30 min, boiled over high heat, and then decocted over low heat for 30 min; then, they were further decocted and concentrated over low heat for 30 min. The liquid was concentrated and evaporated to contain 1 g of raw herbs per mL. The herbs used in the experiments were purchased and prepared at one time and then packed and stored in the refrigerator. The main components of YFJDT were analyzed using high-performance liquid chromatography (HPLC) analysis. Standards were purchased from Shanghai Macklin Biochemical Co., Ltd.

### 2.2. Antibodies

The primary antibodies used in this study included MMP2 (40994, Cell Signaling Technology, Boston, USA), MMP9 (13667, CST, USA), E-cadherin (14472, CST, USA), *β*-cadherin (8480, CST, USA), vimentin (5741, CST, USA), Twist1 (ab50887, Abcam, Cambridge, USA), LC3 II/I (12741S, CST USA), p62 (AF5384, Affinity, USA), PI3K (AF6241, Affinity, USA), p-PI3K (ab32089, Abcam, Cambridge, USA), Akt (4691, CST, USA), p-Akt (5536, CST, USA), p-mTOR (9271, CST, USA), mTOR (2983, CST, USA), GSK3*β* (5676, CST, USA), p-GSK3*β* (9322, CST, USA), PARP (ab32138, Abcam, USA), cleaved-caspase 3 (9664, CST, USA), caspase 3 (9662, CST, USA), cleaved-caspase 8 (8592, CST, USA), caspase 8 (ab108333, Abcam, USA), ATG5 (12994, Abcam, USA), and tubulin (5335, CST, USA).

### 2.3. Tumor-Carrying Mouse Model and Drug Treatment

BALB/c male nude mice (20–24 g) were purchased from Jiangsu Provincial Center for Disease Control and Prevention (animal license number: SYXK (Su)-2017-0030, Jiangsu, China). Nude mice were housed in an IVC environment with free access to feed and pure water. Human A549 cells were purchased from the Cell Bank of the Chinese Academy of Sciences of Shanghai. The cells were cultured in the RPMI-1640 medium in an incubator with a CO_2_ concentration of 5% at 37°C.

A549 cells at logarithmic growth phase were made into a single cell suspension with a cell density of 1 × 10^7^ cells/mL, and 0.2 mL of this suspension was inoculated into the right axillary subcutis of nude mice. A tumor-bearing nude mouse model was successfully developed when the tumor volume of the nude mouse reached 100–150 mm^3^.

Mice were randomly divided into five groups: model group, L-YFJDT group (9 g/kg/d YFJDT), M-YFJDT group (18 g/kg/d YFJDT), H-YFJDT group (36 g/kg/d YFJDT), and gefitinib group (7.5 mg/kg/d gefitinib). Mice in the model group were given the same amount of saline by gavage. The longest diameter and the wide diameter perpendicular of the tumor were measured every 3 days from the first day of administration, and the tumor volume was calculated. After 21 days of treatment, nude mice were euthanized, and the tumors were weighed to calculate the inhibition rate of the YFJDT on the tumors.

### 2.4. Immunohistochemical Detection

The tumors were fixed in 4% formaldehyde and embedded in paraffin and cut into 4 µm thick sections. Sections were incubated with FAT4 antibody (PA5-72970, Invitrogen, USA) for 2 h and incubated with goat anti-rabbit IgG H&L (Alexa Fluor 488) (ab150077, Abcam, USA) for 30 min at room temperature. FAT4 expression rates were determined by using the ImageJ software.

### 2.5. Preparation of YFJDT-Containing Serum

Based on the clinical dose, the effective human dose was calculated to be 2 g/d based on an average adult body weight of 70 kg and 140 mL (g) per day; therefore, YFJDT was administered to SD rats at doses of 12 g/kg/d (according to the clinical dosage × 6). Rats in the low-dose group were given 6 g/kg/d by gavage, rats in the middle-dose group 12 g/kg/d, rats in the high-dose group 24 g/kg/d, and rats in the blank control group an equal volume of distilled water. The blood was collected from the abdominal aorta 1 h after the last YFJDT gavage, and the serum was separated after fasting for 12 h before the last YFJDT gavage. The serum was filtered through a 0.22 microporous filter, sterilized, divided, and stored at −80°C in the refrigerator [15]. The cells were incubated with different concentrations of YFJDT-containing serum for 24 h. The cell proliferation was detected by MTT, and the three drug concentrations that inhibited cell proliferation were screened out.

### 2.6. Cell Culture and YFJDT Treatment

A549 cells were cultured in RPMI-1640 medium containing 10% fetal bovine serum and 1% double antibiotics in a 5% CO_2_ incubator at 37°C. Logarithmic growth phase cells were diluted with serum-free RPMI-1640 medium to a cell count of 5 × 10^5^ to 1 × 10^6^ cells/mL, and 150 *μ*mol/L of CoCl_2_ was used to mimic the hypoxic microenvironment of lung cancer A549 cells. After coincubation of different concentrations of YFJDT-containing serum with cells in a hypoxic environment for 24 h, cell proliferation was detected by the MTT assay, and appropriate drug concentrations that inhibited cell proliferation were screened.

**Experiment 1 .**

To verify the efficacy of YFJDT on A549 cells, cells were divided into four groups: model group, L-YFJDT group, M-YFJDT group, and H-YFJDT group, and all groups were cultured under anoxic environment of CoCl_2_. Cells in each group were cultured with corresponding concentrations of YFJDT-containing serum or not for 24 h to verify the effect of the above-mentioned concentrations of YFJDT on A549 cells.

**Experiment 2 .**

To verify the role of FAT4 in YFJDT-treated A549 cells, cells were divided into model group, H-YFJDT group, FAT4-siRNA group, and FAT4-siRNA + H-YFJDT group, and all groups were cultured under anoxic environment of CoCl_2_. A549 cells or FAT4-siRNA A549 cells were cultured with H-YFJDT-containing serum or not for 24 h to verify the effect of the abovementioned concentrations of YFJDT on A549 cells.

### 2.7. RNA Interference

To silence the FAT4 gene, A549 cells were cultured for 24 h and then transfected with 10 nmol/L targeting FAT4-siRNA referring to the manufacturer's instructions (GeneChem, Shanghai, China). Total RNA from each sample was assayed using RT-PCR to confirm siRNA-induced gene silencing.

### 2.8. Cell Viability Detection

A549 cells were incubated in 5% CO_2_ at 37°C until the cells were plastered and then incubated with the corresponding concentration of YFJDT-containing serum for 24 h. 20 uL of MTT solution was added to each well and incubated for 4 h, and then, 150 uL of dimethyl sulfoxide was added to each well and shaken at low speed for 10 min to dissolve the crystalline material. The absorbance value of each well was measured at OD 490 nm and 570 nm. Therefore, the cell survival rate formula can be expressed as follows: survival rate = (OD_treatment group_/OD_control group_) × 100%.

### 2.9. Cellular Invasiveness Assay

The matrix gel was diluted 1 : 8 and wrapped around the upper chamber surface of the membrane at the bottom of the transwell and air-dried, and the residual culture fluid in the chamber was aspirated. The cells were terminated by digestion and the culture fluid was removed by centrifugation; then, the YFJDT-containing serum-treated cell suspension was adjusted to a cell density of 2 × 10^5^ cells/mL using a serum-free cell culture medium. 200 *μ*L of cell suspension was added to each well of the upper chamber, and 600 *μ*L of culture medium containing 15% fetal bovine serum was added to the lower chamber. The plates were incubated in an incubator at 37°C for 24 h. The chambers were removed and rinsed twice with PBS buffer. The cells on top of the chambers were carefully wiped off with a cotton swab, and the cells on the bottom of the chambers were fixed with 4% paraformaldehyde for 20 min and stained with 0.1% crystal violet for 15 min.

The cell density was adjusted to about 6 × 10^5^ cells/mL, and the cell layer at the bottom of the plate was quickly scratched with a gun along the vertical line of the sterilized ruler, and the 6-well plate was rinsed twice with PBS solution after scratching. After incubation with YFJDT-containing serum for 24 h, the extent of cell healing was observed under the microscope and photographed, and the migration rate was calculated according to the healing of the scratch. Migration rate = ((distance migrated by the intervention group/distance migrated by the control group) − 1) × 100%.

### 2.10. GFP-LC3 Colocalization Assay with Lysosomes

A549 cells in logarithmic growth phase were transfected with the GFP-LC3 plasmid (D2815, Beyotime, Beijing, China). Stably expressed cells were subjected to hypoxia induction after 80% fusion and then incubated for 24 h with the addition of YFJDT-containing serum and a blank control. After incubation, the wells were incubated with 50 nM LysoTracker Red DND-99 (L8010, Solarbio, Beijing, China) for 30 min at 37°C. The cells were washed with PBS and fixed for 1 min and then placed under a fluorescent microscope for observation.

### 2.11. Western Blot Analysis

Equal amounts of protein were separated by SDS-PAGE 12% and transferred onto PVDF membranes. After blocking with 5% skimmed milk for 2 h, the membranes were incubated sequentially with primary antibody and HRP-conjugated secondary antibody. The final protein bands were observed after color development with ECL reagents. The intensity of each sample was determined using the ImageJ software.

### 2.12. Apoptosis Detection by Flow Cytometry

Cells were collected after digestion with EDTA-free trypsin and washed twice with precooled PBS to turn them into a suspension of 5 × 10^6^ cells/mL. YF488-Annexin V (YF®488-Annexin V and PI apoptosis kit, US EVERBRIGHT, China) and 5 *μ*L of PI working solution were added to each tube and incubated on ice for 10–15 min at room temperature, protected from light. 400 *μ*L of PBS was added to each tube and the apoptotic cells were examined by flow cytometry as soon as possible. The fluorescence emission spectrum was detected at 530 nm (FITC channel) and the PI channel emission spectrum at approximately 617 nm.

### 2.13. Quantitative Real-Time Polymerase Chain Reaction

Mixed sample RNA, specific primers, 2X FastKing One-Step RT-PCR MasterMix, and RNase-free double-distilled H_2_O were placed into the real-time PCR instrument using the FastKing One-Step RT-PCR kit (Tiangen, Beijing, China) according to the manufacturer's instructions. The mRNA expression levels of E-cadherin, vimentin, and Twist1 were measured using GAPDH as an internal reference gene. The primers used were as follows: E-cadherin-F, TGGACCGAGAGAGTTTCCCT and E-cadherin-R, CAAAATCCAAGCCCGTGGTG; vimentin-F, TCCGCACATTCGAGCAAAGA and vimentin-R, ATTCAAGTCTCAGCGGGCTC; Twist1-F, CCGTGGACAGTGATTCCCAG and Twist1-R, CCTTTCAGTGGCTGATTGGC; and GAPDH-F, CTGGGCTACACTGAGCACC and GAPDH-R, AAGTGGTCGTTGAGGGCAATG.

### 2.14. Statistical Analysis

The data were analyzed using the GraphPad Prism 8.02 software. Comparisons between groups were made using one-way analysis of variance (ANOVA) and Student's t-test, and values from three independent experiments are expressed as mean ± SD. Differences were considered statistically significant at ^∗^*p* < 0.05 and ^∗∗^*p* < 0.01.

## 3. Results

### 3.1. HPLC Analysis of YFJDT

**Figure 1:**
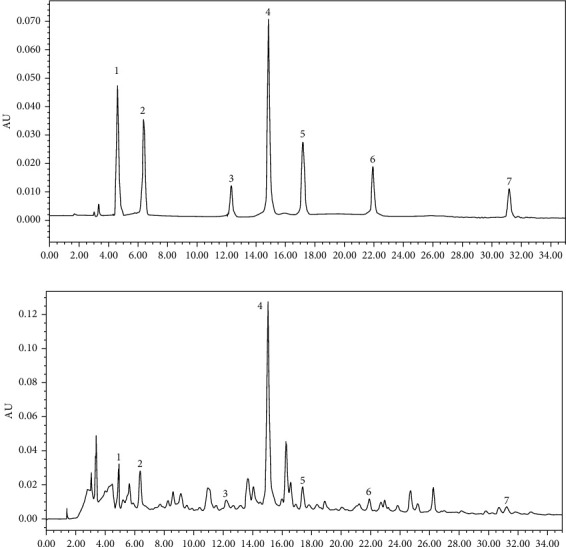
Representative HPLC chromatograms of YFJDT extracts and external standard mix. (a) Chromatographic analysis of 7 standards. (b) Average chromatographic analysis of YFJDT. The 7 common peaks were labelled. 1: chlorogenic acid, 0.857 mg/g; 2: salicylic acid, 0.529 mg/g; 3: militarine, 0.097 mg/g; 4: hyperoside, 1.024 mg/g; 5: rutinum, 0.113 mg/g; 6: wogonin, 0.079 mg/g; 7: psoralen, 0.015 mg/g.

HPLC analysis was performed to evaluate the major component of YFJDT. As shown in Figures 1(a) and 1(b), seven key compounds in YFJDT including chlorogenic acid, salicylic acid, militarine, hyperoside, rutinum, wogonin, and psoralen were determined by the external standard method. The contents of these compounds were 0.857 mg/g, 0.529 mg/g, 0.097 mg/g, 1.024 mg/g, 0.113 mg/g, 0.079 mg/g, and 0.015 mg/g, respectively. Among them, chlorogenic acid, militarine, and hyperoside have been shown to inhibit the proliferation and invasion of A549 and other cancer cells [16–19].

### 3.2. YFJDT Reduces the Volume and Weight of Tumor in Mice

**Figure 2:**
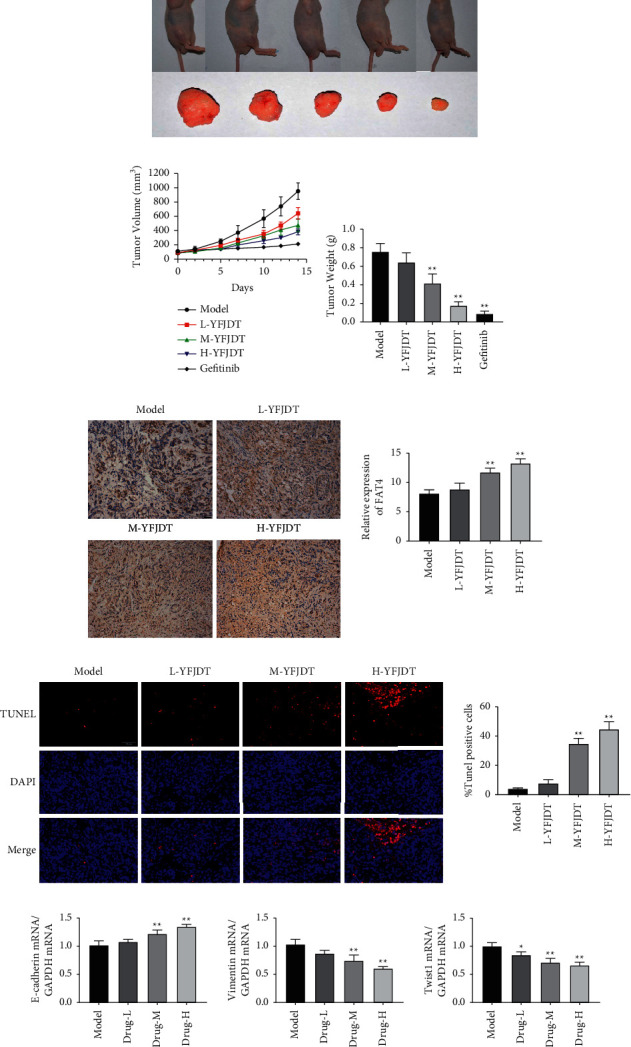
YFJDT reduces the volume and weight of tumor in mice. (a) The size of the tumor in different groups (*n* = 6/group). (b) The volume of the tumor in different groups (*n* = 6/group). (c) The weight of the tumor in different groups (*n* = 6/group). (d) Representative immunofluorescence images of FAT4 in the tumor tissue of different groups. (e) The proportion of apoptosis in the tumor of different groups. (f) The m RNA expressions of E-cadherin, vimentin, and Twist1. Data are expressed as mean ± SD. ^∗^*p* < 0.05 was considered a significant difference compared with the model group, and ^*∗∗*^*p* < 0.01 was considered an extremely significant difference compared with the model group.

To explore whether YFJDT has an antitumor effect in mice, tumor-bearing mice were gavaged with YFJDT and the tumors were photographed and recorded, as shown in Figure 2(a). The volume and weight of the tumors were also recorded, and YFJDT was found to significantly reduce the volume and weight of the tumors (*p* < 0.05, Figures 2(b) and 2(c)). FAT4 has been shown to inhibit epithelial to mesenchymal transition (EMT) and to act as a tumor suppressor [12]. YFJDT treatment increased the content of FAT4 in the lung tumor tissues compared with that of the control group (Figure 2(d)). In Figure 2(f), the mRNA expression of E-cadherin was increased and the mRNA expressions of vimentin and Twist1 were decreased with the treatment of YFJDT. With reference to previous studies, we performed apoptosis assays on tumor tissues and found that the proportion of apoptosis in the tumors of mice in the YFJDT-treated group was greatly increased relative to the model group (*p* < 0.05, Figure 2(e)).

### 3.3. YFJDT Promotes Autophagy-Related Proteins and Inhibits Migration-Related Proteins

**Figure 3:**
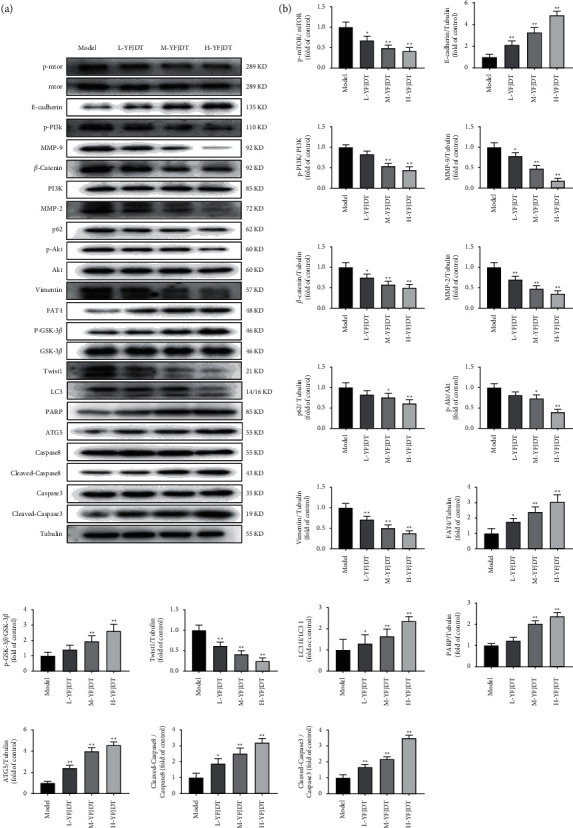
YFJDT promotes autophagy-related proteins and inhibits migration proteins. (a) Western blot analysis of autophagy- and migration-related proteins. (b) Relative expressions of the indicated proteins normalized to levels of tubulin. Data are expressed as mean ± SD. ^*∗*^*p* < 0.05 was considered a significant difference compared with the model group, and ^*∗∗*^*p* < 0.01 was considered an extremely significant difference compared with the model group.

The abovementioned results indicated that YFJDT had an inhibitory effect on lung cancer tumors. As shown in Figures 3(a) and 3(b), the western blot analysis was used to further verify that YFJDT acts by regulating the expressions of proteins. The results showed that treatment with YFJDT increased the expressions of LC3 II/I and E-cadherin and decreased the expressions of MMP2, MMP9, *β*-cadherin, vimentin, Twist1, and p62, while YFJDT also decreased the phosphorylated forms of PI3K, Akt, mTOR, and GSK3*β*, but not PI3K, Akt, mTOR, and GSK3*β* itself.

### 3.4. YFJDT-Containing Serum Inhibits the Proliferation of A549 Cells

**Figure 4:**
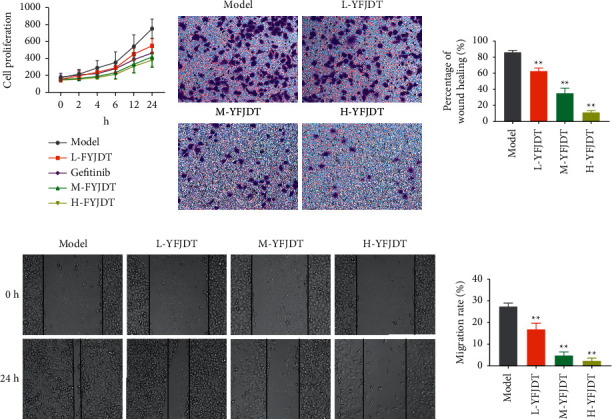
YFJDT-containing serum inhibits the proliferation of A549 cells. (a) The proliferation of A549 cells in each group. (b) The invasion of A549 cells in each group. (c) The migration of A549 cells in each group. Data are expressed as mean ± SD. ^*∗*^*p* < 0.05 was considered a significant difference compared with the model group, and ^*∗∗*^*p* < 0.01 was considered an extremely significant difference compared with the model group.

Cell proliferation was assayed at different times, and it was found that the cell proliferation capacity of the administered group decreased significantly with time compared to the model group in a dose-dependent manner (*p* < 0.05, Figure 4(a)). The efficacy of YFJDT was further validated when we examined the invasive and migratory capacity of YFJDT-treated A549 cells using the transwell assay and cell scratch assay. The transwell migration assay data showed that YFJDT significantly inhibited cell invasion of A549 cells and that higher concentrations of YFJDT-containing serum had a better inhibitory effect than lower concentrations (*p* < 0.05, Figure 4(b)). Similarly, the ability of cell migration was statistically significantly altered by the inhibition of YFJDT (*p* < 0.05, Figure 4(c)).

### 3.5. YFJDT Increases the Expressions of LC3 and FAT4 and the Ratio of Apoptosis

**Figure 5:**
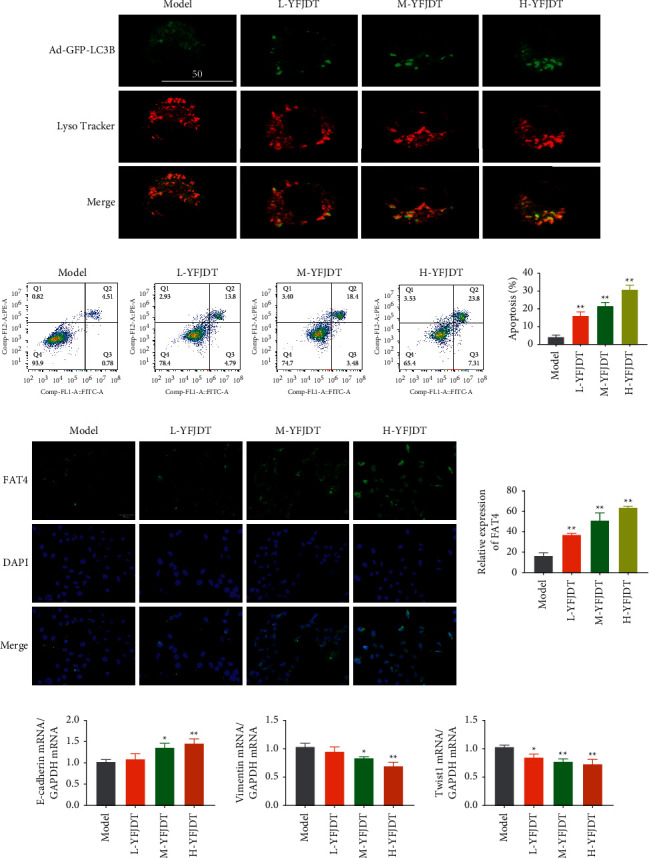
YFJDT increases the expressions of LC3 and FAT4 and the ratio of apoptosis. (a) The colocalization of GFP-LC3 with lysosomes in each group. (b) The apoptosis ratio in A549 cells in each group. (c) Representative immunofluorescence images of FAT4 of A549 cells in each group. (d) The mRNA expressions of E-cadherin, vimentin, and Twist1. Data are expressed as mean ± SD. ^*∗*^*p* < 0.05 was considered a significant difference compared with the model group, and ^∗∗^*p* < 0.01 was considered an extremely significant difference compared with the model group.

To determine whether the decreased viability of A549 cells was associated with autophagy, we examined LC3 to determine the autophagic status of YFJDT-containing serum-treated cells. In the absence of autophagy in A549 cells, the GFP-LC3 fusion protein was diffused in the cytoplasm; in the YFJDT groups, the GFP-LC3 fusion protein translocated to the autophagosome membrane, forming multiple bright green fluorescent spots under fluorescence microscopy, and the colocalization (yellow fluorescence) of GFP-LC3B (green fluorescence) with lysosomes (red fluorescence) increased with increasing drug concentration (Figure 5(a)). Further detection of apoptosis by the flow assay yielded similar results, with higher rates of apoptosis in the YFJDT-treated groups compared to the control group (Figure 5(b)). Interestingly, the contents of FAT4 also increased obviously in YFJDT groups; especially, the highest expression of FAT4 was found in the concentration of YFJDT (*p* < 0.05, Figure 5(c)). In Figure 5(d), the mRNA expression of E-cadherin was increased and the mRNA expressions of vimentin and Twist1 were decreased with the treatment of YFJDT.

### 3.6. YFJDT Promotes Autophagy-Related Proteins and Inhibits Migration-Related Proteins *In Vitro*

**Figure 6:**
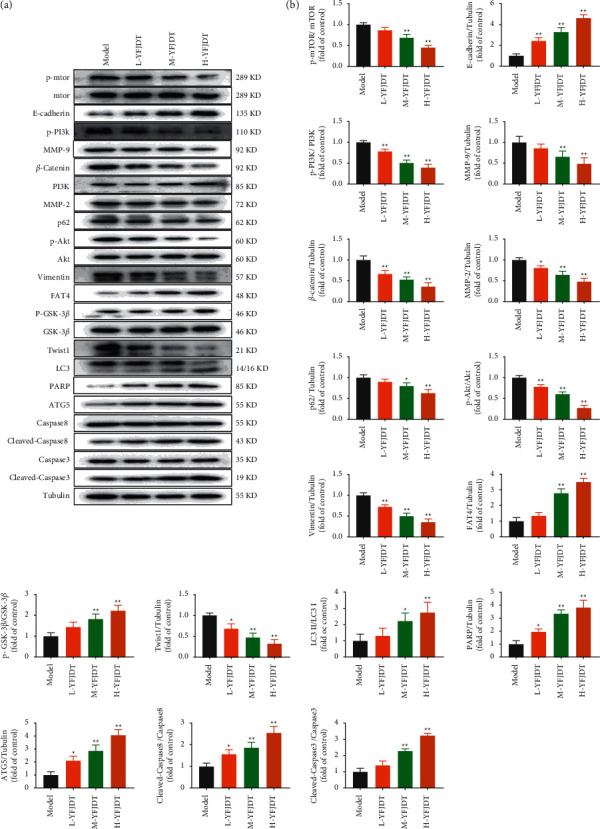
YFJDT promotes autophagy-related proteins and inhibits migration proteins in A549 cells. (a) Western blot analysis of autophagy and migration-related proteins in A549 cells. (b) Relative expressions of the indicated proteins normalized to levels of tubulin. Data are expressed as mean ± SD. ^*∗*^*p* < 0.05 was considered a significant difference compared with the model group, and ^*∗∗*^*p* < 0.01 was considered an extremely significant difference compared with the model group.

Considering the effects of YFJDT on autophagy and migration on A549 cells, the expressions of MMP2, MMP9, E-cadherin, *β*-catenin, vimentin, Twist1, LC3 II/I, p62, p-PI3K, PI3K, Akt, p-Akt, mTOR, p-mTOR, GSK3*β*, and p-GSK3*β* were verified by western blot as shown in Figures 6(a) and 6(b). The results showed that treatment with YFJDT increased the expressions of LC3 II/I and E-cadherin and decreased the expression of MMP2, MMP9, *β*-cadherin, vimentin, Twist1, and p62, while YFJDT also decreased the phosphorylated forms of PI3K, Akt, mTOR, and GSK3*β*, but not PI3K, Akt, mTOR, and GSK3*β* itself.

### 3.7. Si-FAT4 Reduces the Inhibition of YFJDT on the Autophagy and Migration of A549 Cells

**Figure 7:**
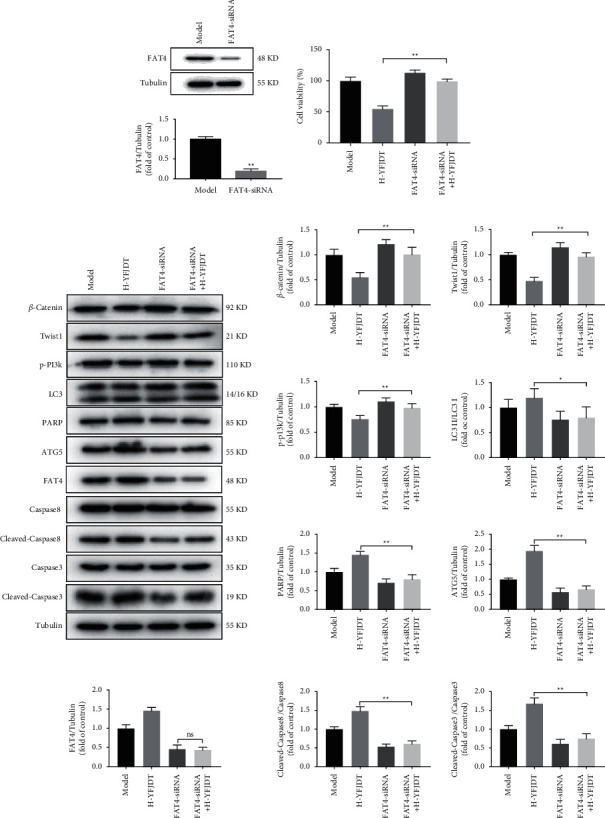
si-FAT4 reduces the inhibition of YFJDT on the autophagy and migration of A549 cells. (a) The expression of FAT4 in A549 cells after treatment with FAT4 si-RNA. (b) The cell viability of A549 cells increased compared to that in the H-YFJDT group. (c) Western blot analysis of autophagy- and migration-related proteins in A549 cells. Data are expressed as mean ± SD. ^*∗*^*p* < 0.05 was considered significant, and ^*∗∗*^*p* < 0.01 was considered extremely significant.

We used FAT4 si-RNA to successfully interfere the expression of FAT4 (Figure 7(a)). At the same time, the decreased cell viability induced by YFJDT compared to the model group has been enhanced in cells treated with the FAT4 si-RNA (Figure 7(b)). Besides these, the YFJDT-induced increase in LC3 expression and the decrease in p-PI3K, Twist1, and *β*-catenin expression were all reversed (Figure 7(c)).

## 4. Discussion

Infiltrative growth and distant metastasis are important biological properties of cancer cells [20]. 90% of cancer deaths are due to migration, and inhibiting tumor migration can slow down tumor progression [21, 22]. Similarly, the majority of NSCLC patients die not due to the primary lesion but due to the consequent advanced metastasis [23]. EMT is the biological process by which epithelial cells are transformed into cells with a mesenchymal phenotype through a specific procedure, mainly manifested by the transformation of the form of an E-cadherin-dominated to vimentin-dominated cytoskeleton [24]. EMT causes epithelial cells to lose their original cell polarity, disrupting the tight junctions of the cell basement membrane and acquiring a high invasive and migratory capacity [25, 26]. Therefore, EMT is an important reason for the enhanced invasive and migratory capacity of epithelial-derived cancer cells.

In our previous study, we observed that YFJDT exhibited interesting antitumor activity against A549 cells [8]. Solid tumor bodies of tumor-bearing mice were exposed to a hypoxic environment, which led to the development of EMT in tumor cells [27]. EMT allows cancer cells to survive independently of the primary tumor site, and these cells may exhibit some degree of sensitivity to autophagy. Based on the important role of tumor cell autophagy in their invasive migration and EMT, we explored whether cellular autophagy is involved in the EMT process and how YFJDT affects this role through the autophagic pathway. To this end, the expressions of autophagy and EMT-related proteins in YFJDT-treated groups and model group were measured. In *in vivo* experiments, YFJDT reduced the volume and mass of the tumor bodies (Figures 2(a)–2(c)). Western blot analysis showed that YFJDT treatment led to an increase in LC3 and E-cadherin and a significant decrease in p62 and E-cadherin, indicating that YFJDT treatment increases autophagy and decreases EMT (Figures 3 and 6). These results demonstrate that YFJDT can inhibit the EMT process in mouse lung cancer tumors, while the exact mechanism needs to be further explored.

FAT4 is a tumor suppressor, and previous studies have shown that FAT4 inhibits EMT and the proliferation of gastric and rectal cancer cells [12,13]. We found that treatment with YFJDT increased FAT4 accompanied by the increase of apoptosis and autophagy and inhibition of EMT, both in tumor-bearing mice and in A549 cells, similar to the previous finding [12]. Twist1 is an important mediator downstream of *β*-catenin, inducing an E-cadherin-mediated decrease in cell-cell adhesion and promoting EMT [28]. The inhibition of EMT by FAT4 may be mediated by levels of *β*-catenin followed by downregulation of Twist1 expression, as confirmed in a previous gastric cancer study [13].

**Figure 8:**
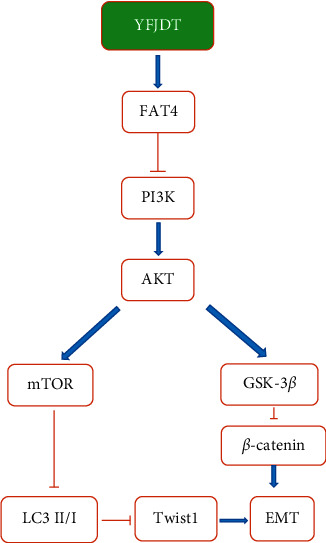
FAT4 can inhibit EMT by promoting autophagy and reducing Twist1 levels.

Cancer cells are exposed to more environmental and intrinsic metabolic stresses than normal cells and may be significantly more dependent on autophagy [29]. In the early stages of cancer cell metastasis, autophagy can inhibit metastasis [7]. In both *in vivo* and *in vitro* experiments, increased FAT4 expression was accompanied by increased expression of LC3 as well as decreased expression of p62. LC3 is a biomarker of autophagy, and the accumulation of p62 reduces autophagy, suggesting that increased FAT4 is accompanied by increased autophagy [29]. Upregulation of autophagy may limit EMT by increasing the instability of key EMT proteins, such as the Twist1 protein [30, 31]. We further interfered FAT4 with FAT4-siRNA and found that the initial inhibition of EMT by YFJDT was blocked in A549 cells (Figure 8).

## 5. Conclusion

We demonstrated for the first time that YFJDT could activate autophagy via FAT4, thereby inducing apoptosis and inhibiting EMT and invasion and migration of A549 cells. However, the further promotion of YFJDT requires statistical and detailed clinical data and herbal toxicity studies and safety evaluation.

## Data Availability

The datasets used and/or analyzed during the current study are available from the corresponding author upon reasonable request.
